# Commentary: Right atrium/inferior vena cava junction—the meeting place

**DOI:** 10.1016/j.xjtc.2021.10.060

**Published:** 2021-11-04

**Authors:** Michael J. Reardon

**Affiliations:** Cardiothoracic Surgery, Houston Methodist DeBakey Heart & Vascular Center, Houston, Tex

**Figure:**
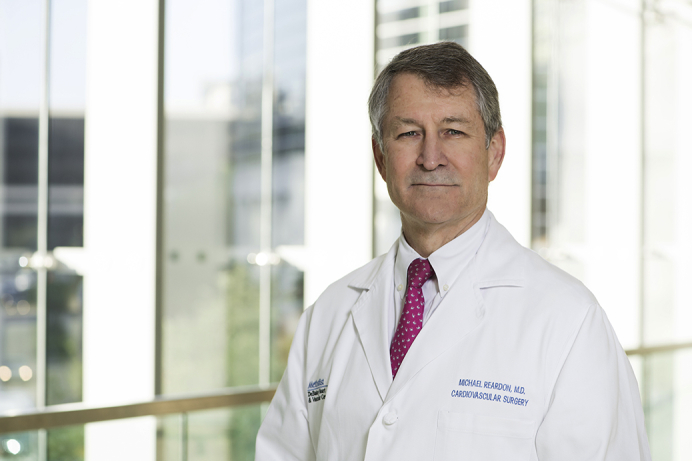
Michael J. Reardon, MD

**Central Message**

A mass obstructing the right atrial inferior vena cava junction requires a multidisciplinary approach to provide optimal evaluation and care.

See Article page 31.

A large obstructing mass at the right atrial/inferior vena cava junction can be both challenging and perplexing. One of the first questions the surgeon must attempt to answer is if this is a tumor or not. Our multidisciplinary cardiac tumor group has extensive experience with large cardiac masses. We have found that cardiac magnetic resonance is very helpful is establishing the presence of perfusion and hence the potential that the mass is a tumor.^1^^,^^2^ If tissue perfusion is seen, we would recommend transvenous biopsy to establish a definitive diagnosis, as primary cardiac sarcoma occurs in this area and neoadjuvant chemotherapy has been shown to improve the rate of negative margin surgical resection as well as survival.^3^ In the absence of perfusion, clot or scar become more likely, and looking for reasons becomes important. Transvenous pacemakers have been seen to cause superior vena cava thrombosis, but this remains asymptomatic due to collateral circulation in 30% to 50% of cases.^4^ Obstruction of the inferior vena cava by a pacemaker lead, however, is rare. Experience with treating this lesion when symptomatic is limited for both endovascular and open surgical approaches.

In this issue of the *Journal*, Smith and colleagues^5^ present a highly unusual case of right atrial/inferior vena cava junction obstruction from a chronic pacemaker lead. An endovascular approach was not successful, and the patient remained highly symptomatic, requiring consideration of an open surgical approach to the problem. The authors organized a multidisciplinary team to address this problem. The chronic pacemaker lead was found to have caused extreme scarring that obstructed the right atrial inflow at the inferior vena cava. Since the pacemaker was no longer needed by the patient, laser lead extraction and open intracardiac removal of the lead was done using cardiopulmonary bypass and cardioplegic arrest. Venous cannulation was via the femoral vein since the superior vena cava was chronically obstructed. The approach used allowed exposure of the right atrium extending to the suprahepatic vena cava at the diaphragm. Using cardioplegic arrest and active suction on the venous cannula, the mass could be seen, resected, and the area patched with bovine pericardium with complete relief of the obstruction. The case is beautifully illustrated and provides an excellent road map for surgeons faced with the need to operate in this area.

There are several cogent issues to highlight. The first is the need to establish a presumed diagnosis of tumor or nontumor. Although rare, masses in this area are generally tumors and not infrequently malignant when they occur. Right atrial sarcoma tends to be bulky, and infiltrative and neoadjuvant chemotherapy makes surgical resection easier, doubles the negative margin, and survival rates.^3^ Advanced imaging will help the surgeon better approach any mass in this area.^2^ The second point to emphasize is the use of a multidisciplinary team to plan and execute treatment. This has been the normal approach in oncology for some time and has recently gained traction for structural heart teams. We have championed the use of multidisciplinary teams for the treatment of cardiac tumors, and the anatomic complexity of this case mimics a complex cardiac tumor.^6^ The excellent outcome seen in this case is a testament to the value of a team approach to complex cardiac masses. The final comment would be to remember that although cardiac pacemakers are common, complications with pacemakers are not infrequent. The right atrial/inferior vena cava junction is an anatomic area in which several specialties meet. Bringing a team to the meeting place to plan and execute treatment provides optimal care, and the Swedish medical group are to be congratulated on their approach and excellent outcome with this complex case.
